# Development and Evaluation of an Ebola Virus Glycoprotein Mucin-Like Domain Replacement System as a New Dendritic Cell-Targeting Vaccine Approach against HIV-1

**DOI:** 10.1128/JVI.02368-20

**Published:** 2021-07-12

**Authors:** Zhujun Ao, Lijun Wang, Hiva Azizi, Titus Abiola Olukitibi, Gary Kobinger, Xiaojian Yao

**Affiliations:** a Laboratory of Molecular Human Retrovirology, Department of Medical Microbiology, Max Rady College of Medicine, Rady Faculty of Health Sciences, University of Manitoba, Winnipeg, Manitoba, Canada; b Centre de Recherche en Infectiologie de l'Université Laval/Centre Hospitalier de l'Université Laval, Québec, Quebec, Canada; Emory University

**Keywords:** Ebola virus glycoprotein, HIV-1, VSV vector, dendritic cells, vaccines, virus-like particles

## Abstract

The development of efficient vaccine approaches against HIV infection remains challenging in the vaccine field. Here, we developed an Ebola virus envelope glycoprotein (EboGP)-based chimeric fusion protein system and demonstrated that replacement of the mucin-like domain (MLD) of EboGP with HIV C2-V3-C3 (134 amino acids [aa]) or C2-V3-C3-V4-C4-V5-C5 (243 aa) polypeptides (EbGPΔM-V3 and EbGPΔM-V3-V5, respectively) still maintained the efficiency of EboGP-mediated viral entry into human macrophages and dendritic cells (DCs). Animal studies using mice revealed that immunization with virus-like particles (VLPs) containing the above chimeric proteins, especially EbGPΔM-V3, induced significantly more potent anti-HIV antibodies than HIV gp120 alone in mouse serum and vaginal fluid. Moreover, the splenocytes isolated from mice immunized with VLPs containing EbGPΔM-V3 produced significantly higher levels of gamma interferon (IFN-γ), interleukin 2 (IL-2), IL-4, IL-5, and macrophage inflammatory protein 1α (MIP-1α). Additionally, we demonstrated that coexpression of EbGPΔM-V3 and the HIV Env glycoprotein in a recombinant vesicular stomatitis virus (rVSV) vector elicited robust anti-HIV antibodies that may have specifically recognized epitopes outside or inside the C2-V3-C3 region of HIV-1 gp120 and cross-reacted with the gp120 from different HIV strains. Thus, this study has demonstrated the great potential of this DC-targeting vaccine platform as a new vaccine approach for improving immunogen delivery and increasing vaccine efficacy.

**IMPORTANCE** Currently, there are more than 38.5 million reported cases of HIV globally. To date, there is no approved vaccine for HIV-1 infection. Thus, the development of an effective vaccine against HIV infection remains a global priority. This study revealed the efficacy of a novel dendritic cell (DC)-targeting vaccination approach against HIV-1. The results clearly show that the immunization of mice with virus-like particles (VLPs) and VSVs containing HIV Env and a fusion protein composed of a DC-targeting domain of Ebola virus GP with HIV C2-V3-C3 polypeptides (EbGPΔM-V3) could induce robust immune responses against HIV-1 Env and/or Gag in serum and vaginal mucosa. These findings provide a proof of concept of this novel and efficient DC-targeting vaccine approach in delivering various antigenic polypeptides of HIV-1 and/or other emergent infections to the host antigen-presenting cells to prevent HIV and other viral infections.

## INTRODUCTION

Despite extensive research and the development of HIV-1 vaccine candidates, to date, only six HIV-1 vaccine efficacy trials have been completed (1, 2). Unfortunately, most of them have shown either no protection or an increased risk for HIV-1 infection. However, the results of the RV144 trial conducted in Thailand, where priming with a canarypox vector (ALVAC), which expressed gag/pol/nef, and boosting with a recombinant HIV gp120 showed modest vaccine efficacy of 31% (3). The outcomes from the RV144 trial strongly support the importance of designing a more effective candidate vaccine against HIV infection.

Dendritic cells (DCs) are the most potent specialized antigen-presenting cells (APCs) that form a critical link between innate and adaptive immune responses (4). Given their central roles in developing immunity, approaches have used DC-based immunotherapy to target HIV-specific antigens to DCs. These approaches have shown the ability to induce various levels of T cell immune responses, such as increased CD4 and CD8 T cell counts, HIV-specific cytotoxic-T-lymphocyte (CTL) responses, and a transient viral load decrease (5–9), as reviewed in reference 10. Therefore, developing new, economically affordable, and effective DC-targeting vaccination strategies may improve the levels of vaccine efficacy.

Ebola virus glycoprotein (EboGP) is the only protein expressed on the Ebola virus (EBOV) surface (11) and prefers to bind with DCs, monocytes, and macrophages (12, 13). Recent studies showed that a vesicular stomatitis virus vector (VSV) expressing the EboGP vaccine was protective against EBOV infections in West Africa with a short time to immunity (14, 15), suggesting that EboGP has strong immunogenicity and may stimulate an effective immune response by itself. Most recently, our reports indicated that the incorporation of EboGP into HIV virus-like particles (VLPs) indeed facilitates DC and macrophage targeting and significantly enhances HIV-specific immune responses (11, 16). This suggests that EboGP has the potential to direct an HIV antigen toward DCs to facilitate effective anti-HIV immune responses. Interestingly, in EboGP, there is a highly glycosylated mucin-like domain (MLD) that encompasses residues 313 to 501 (17) and is located at the apex and the sides of each glycoprotein monomer (18). The MLD has multiple functions during EBOV infection, including protecting conserved regions of the GP and the receptor-binding site from neutralizing antibody recognition (19–21) and masking immune regulatory molecules, such as major histocompatibility complex I (MHC-I), on infected-cell surfaces (20). Interestingly, previous studies have also shown that removing this MLD did not impede EboGP-mediated lentiviral vector entry but enhanced it (22) and that it was dispensable for EBOV infections *in vitro* (17).

HIV-1 envelope glycoprotein (gp160) is required for binding and entry into host cells. The N-terminal subunit, gp120, has a complex fold and can be organized into five conserved regions (C1 to C5) interspersed with five variable areas (V1 to V5). The host CD4 receptor interacts with gp120 to expose the V3 loop, increasing the binding of the V3 loop with CCR5 or CXCR4 chemokine receptors. The GPGR/Q motif at the apex of the V3 loop and at its base is responsible for this binding. Interestingly, human monoclonal antibodies (MAbs) specific for the V3 loop were demonstrated to neutralize various clades of HIV-1 (23–32). Moreover, the RV144 trial results showed that reduced infection was associated with high titers of nonneutralizing IgG antibodies against the V1/V2 region of the envelope protein, while V3-specific antibodies also imposed immune pressure on infecting viruses (10, 33). Thus, the V3 loop is an attractive target for the vaccine to induce a broadly cross-reactive antibody against virus infection.

Based on the above information, we assumed that if we replaced the MLD with heterologous polypeptides, such as the V3 loop derived from HIV gp120, these heterologous polypeptides would also be fully exposed at the apex and the sides of each glycoprotein monomer and be fully exposed to the host immune system to elicit effective immune responses. Thus, in this proof-of-concept study, we replaced the MLD of EboGP with an HIV envelope (Env) polypeptide encompassing the C2-V3-C3 region (EboGPΔM-V3) or C2-V3-C3-V4-C4-V5-C5 loop peptide (EboGPΔM-V3-V5) and demonstrated that VLPs containing the above chimeric proteins could efficiently target human monocyte-derived macrophages (MDMs) and monocyte-derived dendritic cells (MDDCs). Mouse experiments revealed that these VLPs, especially EbGPΔM-V3 VLPs, induced significantly more potent anti-HIV antibodies than wild-type HIV Env VLPs. Specific anti-HIV IgA and IgG antibodies could also be detected in the vaginal fluid of HIV Env/EbGPΔM-V3 VLP-immunized mice. Furthermore, we demonstrated that the coexpression of EbGPΔM-V3 and HIV Env glycoprotein in the rVSV vector elicited robust anti-HIV antibodies that may specifically recognize epitopes outside or inside the V3 region of HIV-1 gp120 and can cross-react with gp120 from different HIV strains (clades B [IIIB0], C [C.1086D7], and AE [AE.A244D11]). Thus, this EboGP MLD replacement expression system can be used as a new approach to improving immunogen delivery to antigen-presenting cells and increasing vaccine efficacy.

## RESULTS

### Generation and expression of EboGPΔM-V3 and EboGPΔM-V3-V5 chimeric fusion proteins.

**FIG 1:**
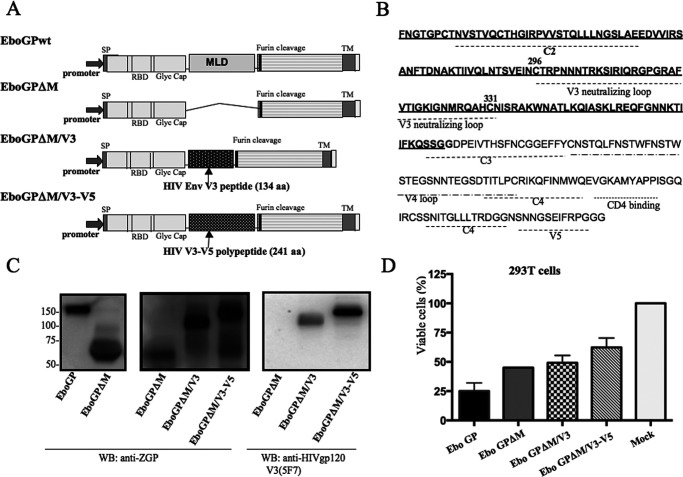
Construction and expression of EboGPΔM-V3 and EboGPΔM-V3-V5 chimeric protein. (A) Codon-optimized EboGP gene and MLD deletion-containing (deletion encompassing aa 306 to 483) EboGP gene sequences were inserted into mammalian cell expression vectors named EboGPwt and EboGPΔM, respectively. PCR-amplified gene sequences including C2-V3-C3 and C2-V3-C3-V4-C4-V5 sequences were inserted into the MLD deletion-containing EboGP gene, and the resulting constructs were designated EboGPΔM-V3 and EboGPΔM-V3-V5. (B) Amino acid sequence of the C2-V3-C3-V4-C4-V5 region of HIV Env. (C) WB detected the expression of EboGPwt, EboGPΔM-V3, and EboGPΔM-V3-V5 in 293T cells by using anti-EboGP (42/3.7) and anti-gp120-V3 (5F7) antibodies. (D) Cytotoxicity of EboGP-based chimeric fusion proteins, detected by the trypan blue exclusion method.

To determine whether the insertion of a heterologous polypeptide(s) in the MLD region of EboGP would affect the cell entry ability of EboGP, we constructed an EboGPΔM-expressing plasmid by deleting the MLD of wild-type EboGP (from amino acids 305 to 485) (Fig. 1A). Furthermore, at the MLD deletion site of EboGPΔM, we inserted a PCR DNA fragment encoding the C2-V3-C3 regions (134 amino acids [aa] of HIV *env*), which contains a V3 loop that bears the GPGR motif (34), or a DNA fragment encoding the C2-V3-C3-V4-C4-V5 regions (241 aa) (Fig. 1B), and the resulting plasmids were named pEboGPΔM-V3 and pEboGPΔM-V3-V5, respectively (Fig. 1A). To examine the expression of each EboGP-HIV Env fusion protein and test whether the glycoproteins were still able to mediate virus entry into susceptible cells, we cotransfected pEboGPwt, pEboGPΔM, a pEboGPΔM-V3 or pEboGPΔM-V3-V5 plasmid with an HIV Gag-Pol packaging plasmid, Δ8.2, and an HIV vector with deletions of multiple genes (reverse transcriptase, integrase, and envelope) (ΔRI/ΔE/GLuc) (35) into 293T cells, as described previously (16, 35). After 48 h of transfection, the expression of fusion proteins, including EboGPwt, EboGPΔM, EboGPΔM-V3, or EboGPΔM-V3-V5, in 293T cells was detected by Western blotting (WB) using a mouse anti-EboGP antibody (Fig. 1C, left and middle). The EboGPΔM-V3 and EboGPΔM-V3-V5 chimeric proteins were recognized by specific anti-gp120 V3-neutralizing antibodies (W0-07 and 5F7) (36) (Fig. 1C, right). These results indicated that HIV gp120 V3 and V3-V5 peptides in the fusion proteins were well expressed and recognized by the anti-gp120 neutralizing antibody.

It was shown previously that EboGP induces cytotoxic effects in human endothelial cells *in vitro* and *in vivo* (37). Hence, we further assessed whether the EboGPΔM-V3 or EboGPΔM-V3-V5 fusion protein could cause a cytotoxic effect in 293T cells. As expected, the expression of EboGPwt resulted in only approximately 30% viable cells, while the viability of EboGPΔM-transfected cells increased to about 50%. Interestingly, there was less cytotoxicity mediated by the EboGPΔM-V3 or EboGPΔM-V3-V5 chimeric protein than by the EboGPΔM protein (Fig. 1D).

**FIG 2:**
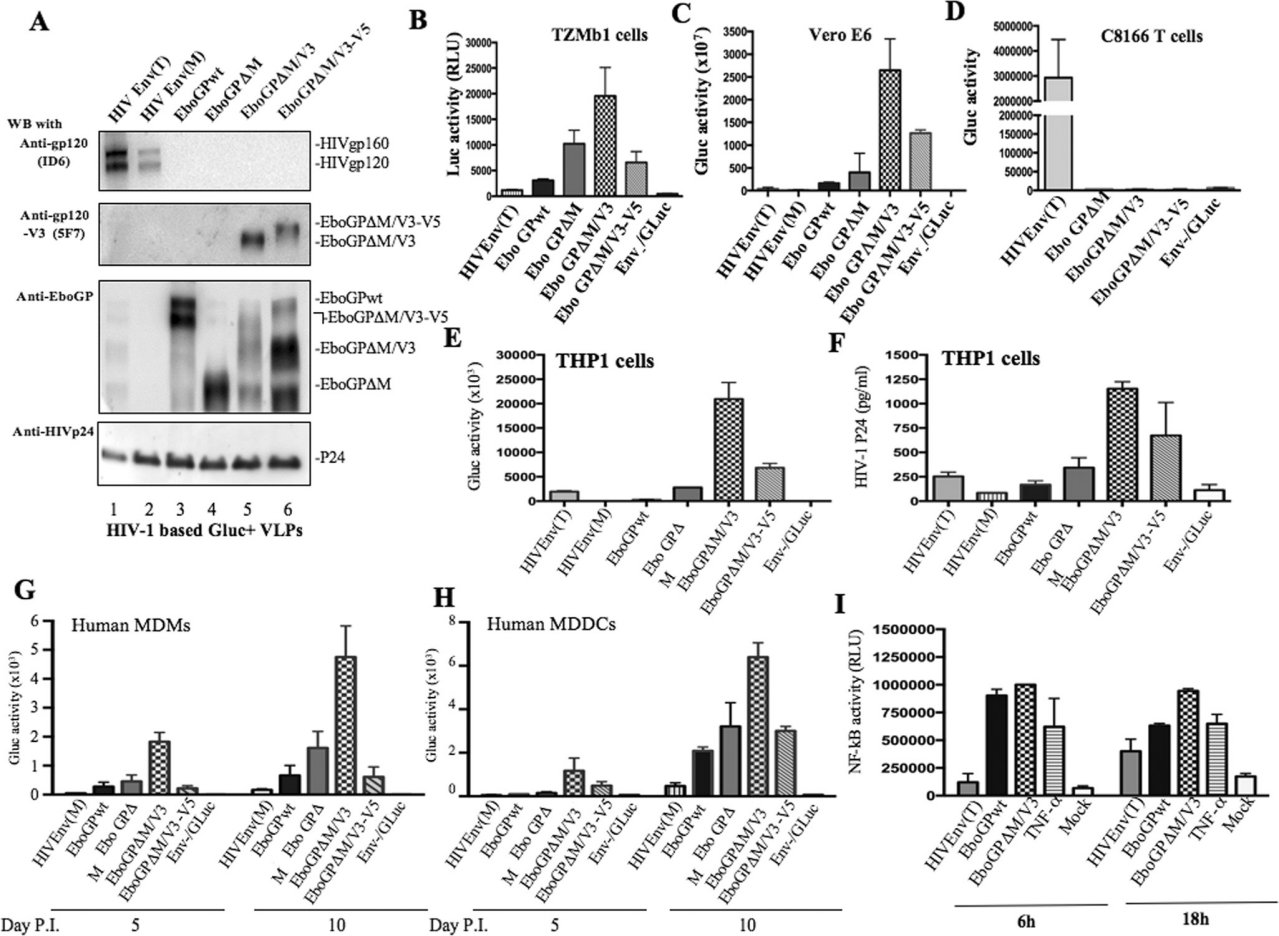
Characterization of the virus entry mediated by HIV Env or EboGP-based chimeric fusion proteins. (A) 293T cells were cotransfected with HIV-1ΔRI/ΔE/GLuc^+^, Δ8.2, and HIV Env (T or M tropic), EboGPwt, EboGPΔM, EboGPΔM-V3, or EboGPΔM-V3-V5 plasmids. The VLPs produced were purified and analyzed by WB with anti-HIV gp120 (ID6 or 5F7), anti-EboGP antibody, or anti-HIV p24 antibody. (B to H) TZM-b1 cells, Vero E6 cells, CD4^+^ C8166 T cells, THP-1 cells, human MDMs, and human MDDCs were infected with the above-described VLPs. At different time points of infection, the supernatants were collected and subjected to a GLuc activity assay or p24 ELISA. (I) The THP1–NF-κB sensor cell line was incubated with various amounts of EboGPwt- or EboGPΔM-V3-pseudotyped HIV VLPs for 6 h to 18 h, and the activation of NF-κB signaling was detected by measurement of the Luc activity of treated cells. TNF-α treatment was used as a positive control. Error bars represent variation between duplicate samples, and the data are representative of results obtained in three independent experiments.

To test whether the insertion of the HIV V3 or V3-V5 fragment would affect the cell entry ability of EboGPΔM, we collected HIV-based VLPs from the supernatants of cotransfected 293T cells as described above. Furthermore, as controls, HIV Env T-tropic (T) or M-tropic (M) VLPs were produced as described previously (16). After purification of the VLPs using ultracentrifugation, we analyzed each VLP stock by WB with mouse anti-EboGP, anti-HIV gp120 (ID6), anti-HIV gp120V3 (5F7), or anti-HIV Gagp24 antibodies. EboGPwt, EboGPΔM, EboGPΔM-V3, and EboGPΔM-V3-V5 was detected as corresponding VLPs (Fig. 2A, third panel, lanes 3 to 6) by the anti-EboGP antibody. As expected, anti-HIV gp120 (ID6), which is directed against the first 204 aa of gp120 (38), could recognize only the full-length HIV Env (Fig. 2A, first panel, lanes 1 and 2), while the anti-HIV gp120V3 (5F7) antibody recognized only the EboGPΔM-V3 and EboGPΔM-V3-V5 fusion proteins (Fig. 2A, second panel, lanes 5 and 6). Similar amounts of the HIV capsid Gagp24 protein were detected in each pelleted VLP (Fig. 2A, fourth panel, lanes 1 to 6).

### Investigation of cell entry mediated by each EboGPΔM fusion protein in various cell lines, primary macrophages, and dendritic cells.

To investigate whether the EboGPΔM fusion protein could mediate cell entry, we used equal amounts of EboGPΔM-, EboGPΔM-V3-, or EboGPΔM-V3-V5-pseudotyped GLuc^+^ VLPs and HIVEnv(T/M)-GLuc^+^ VLPs (adjusted by HIV Gagp24 levels) to infect TZM-b1 cells (HeLa cells expressing CD4^+^/CXCR4 with integrated HIV-LTR-luciferase), a CD4^+^ T lymphoid cell line (C8166 T cells), Vero E6 cells, and a human monocytic cell line (THP1 cells) (39). In parallel, we used a VLP without any viral envelope glycoprotein (Env-GLuc^+^) as a negative control. The results showed that EboGPwt, EboGPΔM, EboGPΔM-V3, or EboGPΔM-V3-V5 VLPs could enter TZM-b1, Vero E6, and THP1 cells effectively and resulted in the expression of HIV-LTR-derived luciferase or GLuc at various levels (Fig. 2B, C, E, and F) but could not enter CD4^+^ C8166 T cells (Fig. 2D). In contrast, HIV Env (T tropic) pseudotyped GLuc^+^ VLPs efficiently infected CD4^+^ C8166 T cells but not Vero E6 cells (compare Fig. 2D and C). Compared with the above-described EboGPwt or EboGPΔM fusion protein, the ability of HIV Env(T)-GLuc^+^ VLPs to enter TZM-b1 and THP1 cells was very low, as expected (Fig. 2B, E, and F). Very interestingly, EboGPΔM/V3 VLPs mediated approximately 3- to 5-fold higher Luc or GLuc activity than EboGPΔM or EboGPΔM-V3-V5 VLPs (Fig. 2B, C, E, and F). All of these data suggest that the replacement of the MLD with the HIV V3 loop fragment in EboGP could increase the cell entry ability of the VLPs in TZM-b1 cells and Vero E6 cells but that VLPs were still unable to enter CD4^+^ T lymphocytes. Notably, both EboGPΔM-V3 and EboGPΔM-V3-V5 VLPs more efficiently infected Vero E6 cells, a U.S. Food and Drug Administration (FDA)-approved cell line for the production of a human vaccine (Fig. 2C). These data suggest that these fusion proteins may be beneficial for industrial viral vector-based vaccine production, such as the production of VLPs and rVSV vectors in Vero E6 cells.

Since EboGP has a preference for targeting DCs and macrophages (12, 13), we next tested whether the insertion of HIV Env V3 or V3-V5 polypeptides could impact the ability of EboGP to infect monocytes and macrophages. The data in Fig. 2G and H clearly show that EboGPwt, EboGPΔM, EboGPΔM-V3, and EboGPΔM-V3-V5 VLPs could more efficiently infect both human MDMs and MDDCs than HIVEnv(M) VLPs without EboGPΔM. Among them, the cell entry ability of EboGPΔM-V3 VLPs was 2- to 3-fold higher than that of EboGPΔM VLPs and EboGPΔM-V3-V5 VLPs (Fig. 2G and H). These data again indicate that the replacement of V3 polypeptides did not interfere with the ability of EboGPΔM to target macrophages but rather enhanced the macrophage-targeting ability of the fusion protein. Overall, these studies provide evidence that the EboGPΔM-V3 fusion has a more efficient ability to target human MDMs and MDDCs.

### EboGPΔM/V3 VLPs could efficiently stimulate the NF-κB signaling pathway.

Previous studies, including ours, revealed that EboGP can induce innate and adaptive immune responses by stimulating human DCs through the NF-κB and mitogen-activated protein kinase (MAPK) signaling pathways (16). Since our above observations showed that EboGPΔM-V3 displayed an efficient ability to target MDMs and MDDCs, we further checked the potential of EboGPΔM-V3 to activate the NF-κB signaling pathway. A THP-1–NF-κB sensor cell line utilizing an NF-κB–Cignal Lenti luciferase reporter system was generated as described previously (16). THP-1–NF-κB sensor cells were treated with equivalent amounts of HIV Env-, EboGPwt- or EboGPΔM-V3 VLPs, and THP-1–NF-κB sensor cells were treated with tumor necrosis factor alpha (TNF-α) as a positive control. After 6 h and 18 h, the cells were lysed, and the Luc activity in the cells was measured to determine the activation levels of the NF-κB signaling pathways. We found that HIV Env VLP treatment resulted in a modest increase in Luc activity after 6 h of stimulation compared to that in nontreated cells (Fig. 2H). However, both EboGPwt VLPs and EboGPΔM-V3 VLPs induced higher levels of Luc activity as early as 6 h after treatment, and the EboGPΔM-V3 VLP-treated cells maintained the most increased NF-κB activity until 18 h (Fig. 2I), suggesting that the EboGPΔM-V3 VLPs have a solid ability to stimulate the NF-κB pathway in THP-1–NF-κB sensor cells.

### EboGPΔM-V3-pseudotyped VLPs induced elevated IL-6, IL-10, MIP-1α, and TNF-α gene expression in human MDMs.

**FIG 3:**
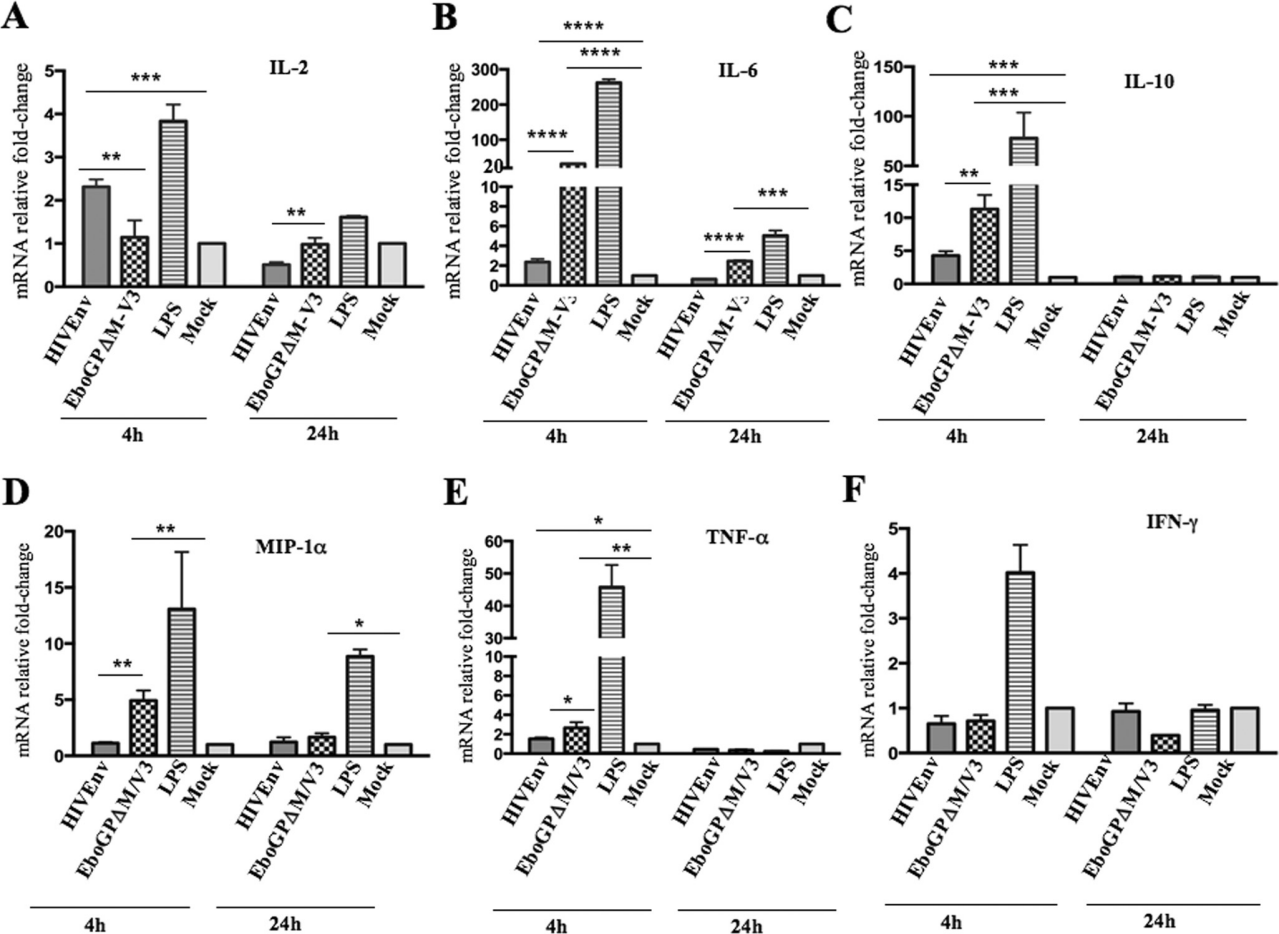
Gene expression of proinflammatory cytokines and chemokines induced by HIV Env or EboGPΔM-V3 VLPs in human MDMs. Human MDMs were treated with HIV Env or EboGPΔM-V3 VLPs for 4 or 24 h. LPS treatment was included as a control. The intracellular levels of mRNA transcripts encoding IL-2, IL-6, IL-10, MIP-1α, IFN-γ, and TNF-α (A to F) were analyzed by quantitative real-time PCR. Error bars represent variation between samples from three donors. Statistical significance was determined using an unpaired *t* test. *, *P* ≤ 0.05; **, *P* ≤ 0.01; ***, *P* ≤ 0.001; ****, *P* ≤ 0.0001.

Previous studies have shown that Ebola virus VLPs or Ebola virus GPs can activate macrophages and produce cytokines and chemokines through the Toll-like receptor 4 (TLR4) signaling pathway (40–45). In this study, we wanted to evaluate and compare the proficiencies of EbGPΔM-V3 VLPs and HIV Env VLPs in differentially regulating cytokine or chemokine gene expression in human MDMs. Briefly, human primary MDMs were treated with the same amounts of HIVEnv(M) VLPs or EboGPΔM-V3 VLPs. At 4 and 24 h postexposure, the intracellular levels of several cytokine and chemokine mRNA transcripts were measured using quantitative real-time PCR. In parallel, lipopolysaccharide (LPS) treatment was included as a positive control. The results revealed that at 4 h after treatment, HIVEnv(M) VLPs alone induced a significant increase in interleukin 2 (IL-2), IL-6, IL-10, and TNF-α (1.5- to 5-fold), but levels decreased dramatically at 24 h (Fig. 3A, B, C, and E). Interestingly, treatment with EboGPΔM-V3 VLPs induced significantly higher mRNA levels of IL-6, IL-10, macrophage inflammatory protein 1α (MIP-1α), and TNF-α than HIVEnv(M) VLP after 4 h of treatment (Fig. 3B to E). However, neither HIV Env nor EboGPΔM-V3 VLP treatment increased gamma interferon (IFN-γ) mRNA expression in MDMs (Fig. 3F). We also tried to detect the levels of different cytokines and chemokines in the supernatant, which was unsuccessful, possibly due to the lower sensitivity of the assay (data not shown). Overall, gene expression analyses indicated that after exposure to EboGPΔM-V3 VLPs, human MDMs induced more profound IL-6, IL-10, MIP-1α, and TNF-α gene expression than HIV Env(M) VLPs.

### EboGPΔM-V3 VLP immunization induced significantly higher levels of anti-HIV IgA, IgG, and cytokine/chemokine production.

**FIG 4:**
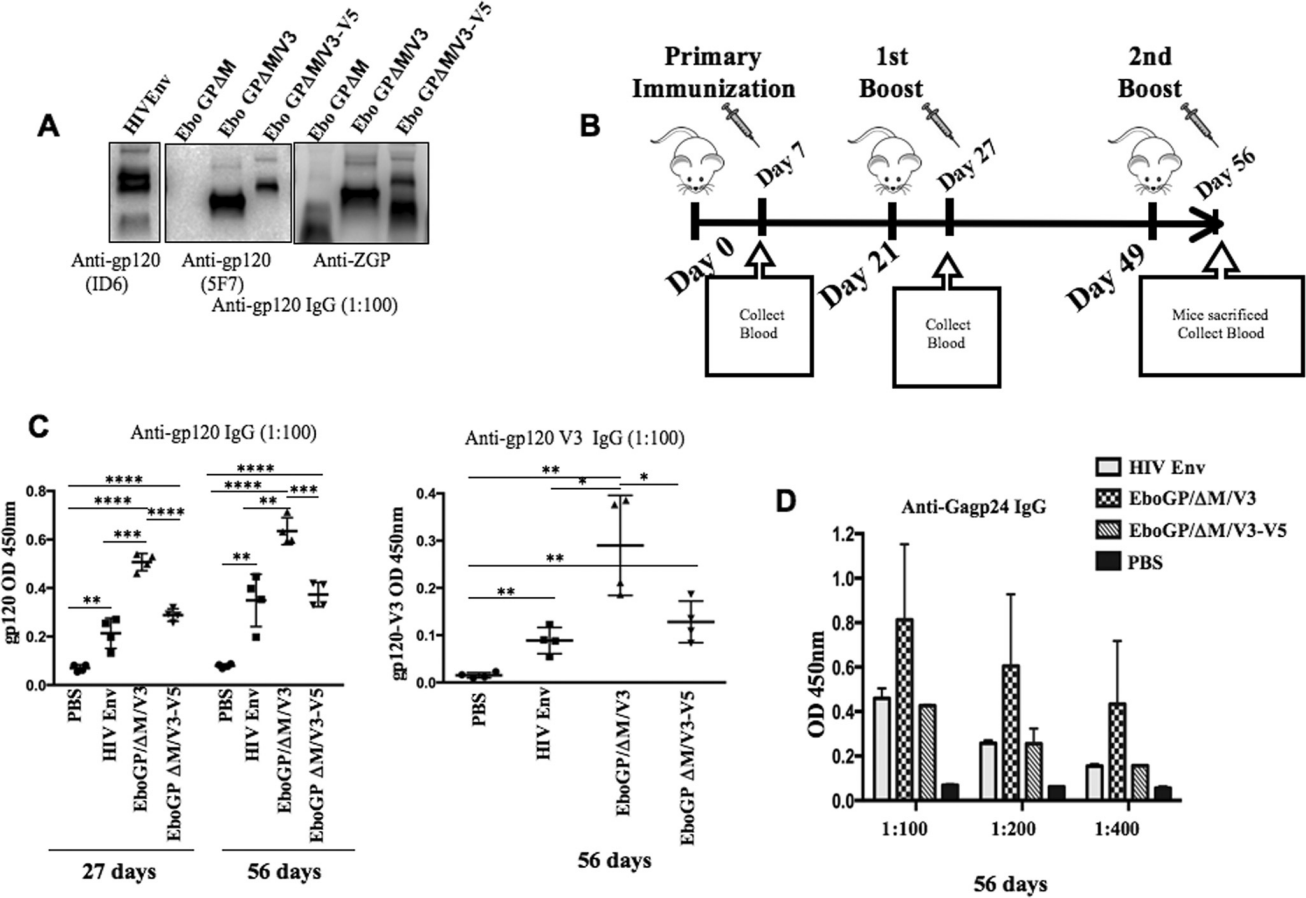
Higher levels of anti-HIV antibodies induced by EboGPΔM-V3-pseudotyped VLPs in BALB/c mice. (A) Detection of the presence of HIV Env, EboGPΔM-V3, or EboGPΔM-V3-V5 in the VLPs used for immunization. (B) BALB/c mice were injected subcutaneously with 100 ng (p24) of HIV Env, EboGPΔM-V3, or EboGPΔM-V3-V5 VLPs on day 0 as indicated. At 21 and 49 days postimmunization, mice were given boosters with the same amounts of VLPs. Sera were collected at 7, 27, and 56 days postimmunization. (C) Levels of anti-HIV gp120 IgG (left) and anti-gp120 V3 IgG (right) in mouse sera. (D) Anti-Gagp24 antibodies in mouse sera were detected via ELISA. Statistical significance was determined using an unpaired *t* test. *, *P* ≤ 0.05; **, *P* ≤ 0.01; ***, *P* ≤ 0.001; ****, *P* ≤ 0.0001.

Since EboGPΔM-V3 VLPs could mediate efficient virus entry in MDDCs/MDMs and generate higher levels of several cytokines and chemokine mRNAs in MDMs, we next investigated whether the incorporation of EboGPΔM-V3 and EboGPΔM-V3-5 into HIV VLPs could enhance the immunogenicity of VLPs *in vivo*. As depicted in Fig. 4A and B, we first subcutaneously immunized BALB/c mice with 100 ng (p24) of HIV Env-, EboGPΔM-V3-, or EboGPΔM-V3-V5-pseudotyped HIV VLPs on days 0, 21, and 49. On days 7, 27, and 56 postimmunization, mouse sera were collected, and anti-HIV gp120 and anti-HIV p24 antibody levels were measured by enzyme-linked immunoassay (ELISA) against recombinant gp120 (IIIB) and p24 proteins. The results revealed that the titers of HIV-specific humoral immune responses against HIV Env gp120 and Gagp24 from mice immunized with EboGPΔM-V3 VLPs were higher than those of HIV Env VLPs at both 27 and 56 days (Fig. 4C and D). However, the analysis revealed similar anti-HIV gp120 or anti-p24 antibody production levels in EbGPΔ-V3-V5 VLP- and HIV Env VLP (Henv VLP)-immunized mice (Fig. 4C, left, and Fig. 4D). We further assessed the ability of Henv-, EbGPΔM-V3- or EboGPΔM-V3-V5 VLPs to induce anti-V3 Abs in the sera. The sera of all mice were tested for reactivity with an HIV-1 consensus B V3 peptide (CKSIHIGPGRAFYTTGC). The data in Fig. 4C (right) indicate that EboGPΔM-V3 VLPs can significantly induce the immune response to the V3 epitope. These results provide evidence that the presence of EbGoPΔM-V3 alone in VLPs induced broad and significantly higher immune responses against HIV gp120 and Gagp24 in immunized mice.

**FIG 5:**
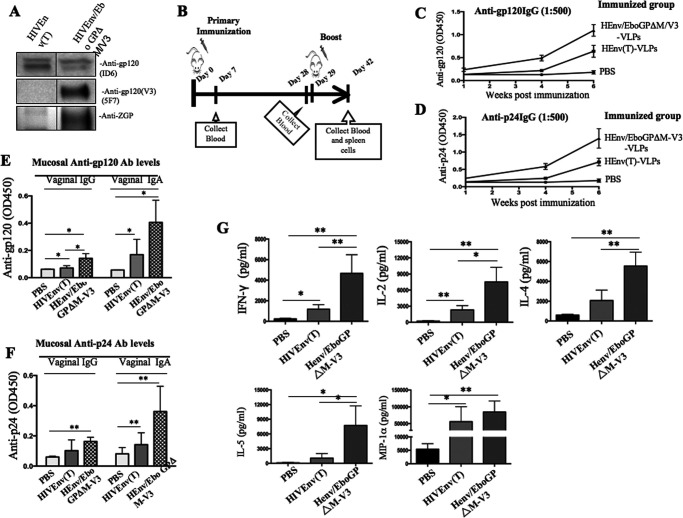
Significantly higher levels of anti-HIV antibodies and cytokines/chemokines were induced by immunization with HIV Env/EboGPΔM-V3-pseudotyped VLPs in BALB/c mice. (A) WB detected the presence of HIV Env and EboGPΔM-V3 in the pseudotyped VLPs. (B) BALB/c mice were injected subcutaneously with 100 ng (p24) of HIV Env or HIV Env/EboGPΔM-V3 VLPs and given boosters with the same amounts of VLPs at day 29. Sera were collected at 7, 28, and 42 days postimmunization. The levels of anti-HIV gp120 (C) and anti-HIV p24 (D) antibodies in sera were detected via ELISA at the time points indicated. The levels of anti-HIV gp120 IgG and IgA (E) and anti-HIV p24 IgG and IgA in vaginal (F) were detected via ELISA. Error bars represent variation between samples of each immunized group. (G) Splenocytes isolated from immunized mice were stimulated with HIV Env peptides. After 72 h of stimulation, supernatants were collected, and the release of cytokines and chemokines in the supernatants was quantified with an MSD V-plex kit mouse cytokine kit and counted in the MESO Quickplex SQ120 instrument. Statistical significance between the two groups was determined using an unpaired *t* test. *, *P* ≤ 0.05; **, *P* ≤ 0.01.

In the next mouse experiment, we tested whether the co-incorporation of HIV Env with EbGPΔM-V3 (Henv/EbGPΔM-V3) into VLPs could enhance the humoral immune response. To this end, we immunized BALB/c mice with 100 ng (p24) of Henv- or Henv/EboGPΔM-V3-pseudotyped VLPs on days 0 and 29. At day 42 postimmunization, mouse sera were collected, and anti-HIV gp120 and anti-HIV p24 antibody levels were measured by the corresponding ELISA (Fig. 5A and B). Interestingly, Henv/EboGPΔM-V3 VLPs elicited a much stronger humoral response against both HIV-Gagp24 and HIV-Gagp120 than HIV Env alone (Fig. 5C and D). These results suggest that VLPs pseudotyped with both EboGPΔM-V3 and HIV Env have a very strong ability to stimulate immune responses.

Given that mucosal surfaces are a primary route of HIV-1 infection, we evaluated the levels of specific anti-HIV gp120 and anti-p24 IgA and IgG in mouse vaginal fluids after 42 days of immunization with Henv VLPs or Henv/EboGPΔM-V3 VLPs. The results showed that higher levels of anti-HIV-Gagp24 and HIV gp120 IgA and IgG were detected in the vaginal secretion fluids from Henv/EboGPΔM-V3 VLP-immunized mice than in those from Henv VLP-immunized mice (Fig. 5E and F). The control mouse group, which received only phosphate-buffered saline (PBS) injection, did not generate any HIV-specific antibodies. These results indicate that the immunization of Henv/EboGPΔM-V3 VLP-immunized mice can induce significantly stronger humoral immune responses, including on the vaginal mucosal surface.

We next evaluated the cell-mediated immune responses of HIV VLP-immunized mice against HIV Env. Splenocytes isolated from HIV Env- or Henv/EboGPΔM-V3 VLP-immunized BALB/c mice were stimulated with HIV Env peptides, and the released cytokines and chemokines were quantified using a MSD V-plex mouse cytokine kit, as described in Materials and Methods. Results indicated that the splenocytes isolated from Henv/EbGPΔM-V3 VLP-immunized mice produced significantly higher levels of IFN-γ, IL-2, IL-4, IL-5, and MIP-1α than splenocytes from PBS-treated mice (Fig. 5G). Also, HIV Env VLPs immunization induced clearly higher levels of IFN-γ, IL-2, and MIP-1α than in the splenocytes from PBS-treated mice. Interestingly, results also revealed that after HIV Env peptide stimulation, the splenocytes from Henv/EboGPΔM-V3 VLP-immunized mice achieved approximately 2- to 3-fold-higher levels of IFN-γ, IL-2, IL-4, and IL-5 than HIV Env VLP-immunized mice splenocytes (Fig. 5G). Overall, these results depicted a more substantial stimulating effect of cellular immunity in the mice immunized with Henv/EboGPΔM-V3 VLPs.

### rVSV-coexpressing Henv/EboGPΔM-V3 chimeric protein induced robust anti-HIV gp120 humoral responses.

**FIG 6:**
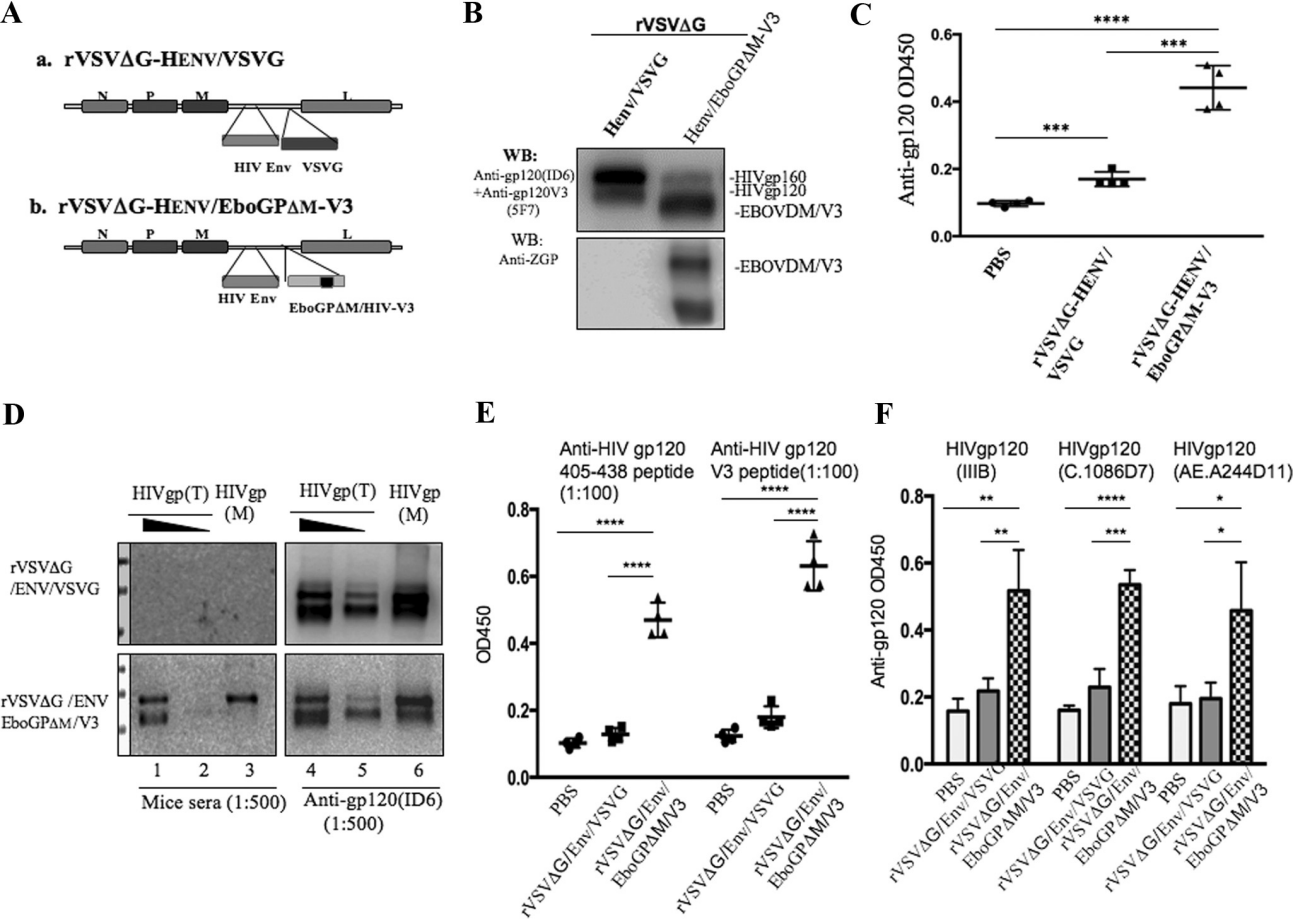
Generation of rVSV coexpressing HIV Env and EboGPΔM-V3 and induction of potent anti-HIV immune responses in mice. (A) Schematic structures of the rVSV vector coexpressing HIV Env and VSV-G (rVSVΔG/Henv/VSVG) and rVSV coexpressing HIV Env and EboGPΔM-V3 (rVSVΔG/Henv/EboGPΔM-V3). (B) WB detected the expression of HIV Env and EboGPΔM-V3 in the pseudotyped rVSV by using anti-HIV gp120, anti-HIV gp120V3 (top), or anti-ZGP (bottom). (C) The anti-HIV gp120 antibody levels in the sera of immunized mice were measured by anti-HIV gp120 IgG ELISA. The value is the individual amount for each mouse of each group. (D) The mouse serum pool from the rVSVΔG/Henv/VSVG and rVSVΔG/Henv/EboGPΔM-V3 groups was analyzed by WB against lysates from cells transiently expressing T- or M-tropic HIV Env protein (left). Meanwhile, the monoclonal anti-gp120 antibody (ID6) was used to detect HIV Env protein as a control (right). (E) ELISA was also done with the HIV-1 consensus B V3 peptide or HIV Env 405–423 and 419–433 peptides. (F) Cross-clade reactivity of anti-HIV gp120 antibodies from immunized mice. HIV gp120 recombinant proteins derived from clades B (IIIB0), C (C.1086D7), and AE (AE.A244D11) were used for ELISA to detect the cross-reactivity of the mouse serum pool from each group. Error bars show variation between triplicate samples of each group. Statistical significance was determined using an unpaired *t* test. *, *P* ≤ 0.05; **, *P* ≤ 0.01; ***, *P* ≤ 0.001; ****, *P* ≤ 0.0001.

Since the recombinant VSV (rVSV) vector is a replication competent vaccine and has been shown to induce both strong humoral and cell-mediated immune responses (46), we further investigated whether the coexpression of EboGPΔM-V3 and the HIV Env glycoprotein in the recombinant rVSV vector could also enhance the anti-HIV immune response *in vivo*. First, two rVSV vectors (Fig. 6A), including rVSV coexpressing HIV envelope glycoprotein and EboGPΔM-V3 (rVSVΔG/Henv/EboGPΔM-V3) and rVSV coexpressing HIV envelope glycoprotein and VSV-G (rVSVΔG/Henv/VSVG) (Fig. 6A), were rescued in Vero E6 cells by using a reverse genetics technique (47). After rVSVΔG/Henv/EboGPΔM-V3 and rVSVΔG/Henv/VSVG were produced in Vero E6 cells, the expression of HIV envelope glycoprotein and EboGPΔM-V3 was detected in Vero E6 cells (Fig. 6B). Then, BALB/c mice were injected intraperitoneally with 1 × 10^6^ 50% tissue culture infective doses (TCID_50_) of rVSVΔG/Henv/VSVG or rVSVΔG/Henv/EboGPΔM-V3. At 35 days postimmunization, the sera from immunized mice were collected and measured for levels of anti-HIV gp120 antibody against recombinant HIV gp120 (IIIB) by ELISA. The results showed that rVSVΔG/Henv/EboGPΔM-V3 elicited significantly higher anti-HIV gp120 antibody responses than rVSV vectors coexpressing HIV Env and VSV-G (Fig. 6C). Consistent with this, by using WB, we confirmed that the serum (at a 1:500 dilution) from the mice immunized with rVSVΔG/Henv/EboGPΔM-V3 strongly recognized both the T- and M-tropic forms of HIV Env proteins (Fig. 6D, bottom left). In contrast, the sera (at the same dilution) from mice immunized with HIV Env/VSVG did not react with Env proteins (Fig. 6D, top left), even though similar amounts of Env proteins were detected by a monoclonal anti-gp120 antibody (Id6) (Fig. 6D, right). To determine whether the antibodies raised from rVSVΔG/Henv/EboGPΔM-V3-immunized mice were only induced by the V3 region, which was fused with EboGPΔM, we tested the specificity of serum with ELISA using plates coated with an HIV-1 consensus B V3 peptide (CKSIHIGPGRAFYTTGC) or the peptide mix of HIV Env 405–423 and 419–433, which are regions outside V3 of gp120. As shown in Fig. 6E, mice immunized with rVSVΔG/Henv/EboGPΔM-V3 exhibited much stronger antibody responses against both the V3 peptide and the 405–433 and 419–433 regions of gp120 than mice immunized with rVSVΔG/Henv/VSVG. These results clearly indicated that the presence of EboGPΔM-V3 fusion proteins in rVSV/Henv resulted in a robust anti-HIV Env immune response that targeted not only the V3 region but also other regions of HIV gp120.

To assess whether the anti-HIV-Env serum collected from immunized mice could cross-reactivate with gp120 from other HIV strains, HIV gp120 recombinant proteins derived from clade B (IIIB0), clade C (C.1086D7), and clade AE (AE.A244D11) were used for ELISA, as described in Materials and Methods. The results revealed that the sera from rVSVΔG/Henv/EboGPΔM-V3-immunized mice were able to recognize these three clades of gp120 with a significantly higher affinity (Fig. 6F), indicating that rVSVΔG/Henv/EboGPΔM-V3 induces anti-HIV antibodies with broad cross-reactivity.

## DISCUSSION

In this study, we describe a novel EboGPΔM-based fusion protein vaccine technology in which a large polypeptide encompassing the C2-V3-C3 regions (134 aa) or C2-V3-C3-V4-C4-V5-C5 polypeptide (243 aa) of HIV Env was fused with an MLD deletion-containing EboGP (EboGPΔM). The results demonstrated that the EboGPΔM-V3 and EboGPΔM-V3-V5 fusion proteins were able to mediate efficient virus entry into human APCs, including MDMs and MDDCs. Furthermore, HIV VLPs or rVSV coexpressing HIV Env and EboGPΔM-V3 elicited robust host humoral immune responses against both HIV gp120 and Gagp24 in serum and vaginal mucosa. This technology demonstrates the feasibility of inserting various large heterologous polypeptides into EboGPΔM as novel DC-targeting vaccine approaches for various viral infections, including HIV-1 infection.

We recently reported that incorporating either EboGPwt or a mucin-like domain deletion mutant, EboGPΔM, into HIV VLPs significantly facilitates dendritic cell/macrophage targeting and enhances HIV-specific immune responses, indicating that EboGP has a strong ability to direct HIV virus-like particles toward APCs and promote effective immune responses (16). Given that the MLD of EboGP is located at the apex and the sides of each EboGP monomer, we assumed that if we were able to insert various heterologous polypeptides at the MLD deletion region, these immunogenic polypeptides would also be very efficiently exposed to the host immune system to elicit effective immune responses. Thus, we tested this hypothesis by inserting two peptides (V3 and V3-V5 peptides) derived from HIV (pNL4.3) envelope glycoprotein (Env) into EboGPΔM (Fig. 1A and B). Our studies clearly indicated that neither fusion protein disrupted the cell entry ability of EboGPΔM (Fig. 2). These observations demonstrated that EboGPΔM is able to hold a large peptide(s), such as HIV V3 peptide (134 aa) or HIV V3-V5 (243 aa), in the position of MLD while maintaining its effective CD/MDM-targeting and cell entry abilities. Thus, these findings provide proof of concept for a DC-targeting EboGPΔM-based fusion protein technology as a novel vaccine approach to deliver large heterologous polypeptides for various vaccination developments.

VLPs are ideal immunogens, since they mimic the morphology of pathogens and display antigens in their native conformations. In this study, EboGPΔM-V3 or EboGPΔM-V3-V5 fusion protein-pseudotyped HIV VLPs were first tested for their immunogenicity in mice, and the results revealed that the presence of EboGPΔM-V3 in VLPs correlated with significantly higher and broad anti-HIV immune responses than native HIV Env VLPs (Fig. 4). Obviously, this is due to the presence of the EboGPΔM-V3 fusion protein in VLPs, which renders them more efficiently targeted (Fig. 2G and H) and activates DCs/macrophages (Fig. 2I). The EboGPΔM-V3-V5 VLPs induced a lower immune response than EboGPΔM-V3 (Fig. 4C and D). This may be due to the facts that EboGPΔM-V3-V5 was less efficiently incorporated into pseudovirus than EboGPΔM-V3 (Fig. 4A) and/or that EboGPΔM-V3-V5-pseudotyped virus displays a lower ability to enter DCs/macrophages (Fig. 2G and H). Since the establishment of specific anti-HIV immune responses, particularly at mucosal sites during vaccination, is an essential parameter for successful HIV vaccine development, we further evaluated the specific anti-HIV gp120, anti-p24 IgA, and IgG levels in the vaginal fluids of immunized mice. Interestingly, the analyses indicated that immunization with the Henv/EboGPΔM-V3 VLPs induced significantly higher anti-HIV gp120 and anti-p24 IgA and IgG antibody levels in vaginal fluid. These observations demonstrated the importance of the DC/MDM-targeting feature of EboGPΔM as a vaccine strategy for eliciting a significant and broad immune response.

We also asked whether the V3 loop of gp120 in EboGPΔM-V3 would lead to a dominant immune response, since previous studies have reported the majority of the immune responses to target epitopes in the more variable regions of HIV Env (48–50). In other words, HIV V3 in the MLD position serves as an immunodominant “decoy” that may absorb immune reactivity and potentially preclude responses against other protective epitopes. To answer this question, we performed an ELISA by using a plate coated with the GPGR V3 crown region peptide (CKSIHIGPGRAFYTTGC) or V4-C4 (aa 405 to 433) peptide, located outside the C2-V3-C3 loop. We noticed that mice immunized with rVSVΔG/Henv/EboGPΔM-V3 reacted to both the V3 peptide and V4-C4 peptides (Fig. 6E), suggesting that high exposure of the V3 region of rVSVΔG/Henv/EboGPΔM-V3 did not induce an immunodominant decoy effect. These results also provide evidence for the feasibility of fusing HIV Env conserved regions, such as MPER, which comprises the last 24 C-terminal amino acids of the HIV-1 gp41 ectodomain (51), with EboGPΔM to induce a potent and broad immune response against different subtypes of HIV strains, which are under investigation.

It is well known that both innate and adaptive immune responses are essential for vaccine-induced protection. Several *in vitro* and animal studies have shown that EboGP alone or EboGP-pseudotyped VLPs can stimulate monocytes and macrophages to induce a strong innate immune response, including the production of several inflammatory cytokines and chemokines such as IL-1β, IL-6, IFN-γ, and TNF-α (40, 42–45, 52). In agreement with previous studies, we showed that exposure of human macrophages to EboGPΔM-V3 VLPs was able to stimulate NF-κB pathways (Fig. 2I). Consequently, elevated expression of cytokines and chemokines, including IL-6, IL-10, TNF-α, and MIP-1α was observed at 4 h after exposure (Fig. 3). This indicates that the EboGPΔM-V3 fusion protein can efficiently mediate VLP entry into DCs/MDMs and activate MDM innate immune responses. Furthermore, our animal studies also revealed the stronger stimulatory effect of Henv/EbGPΔM-V3 VLPs on the production of cytokines and chemokines, including IFN-γ, IL-2, IL-4, IL-5, and MIP-1α, in the immunized mice (Fig. 5G). These results clearly indicate that immunization with Henv/EbGPΔM-V3 VLP can induce a stronger cellular immunity.

The finding that the MIP-1α gene expression was significantly elevated after human MDMs being treated or mice being immunized, respectively, with EboGPΔM-V3 VLPs (Fig. 3D and 5G) agrees with our recent observation that Henv/EboEPwt- or Henv/EboEPΔM VLP-immunized mouse splenocytes produced significantly higher levels of MIP-1α (16). EboGPΔM-V3-mediated increased expression of MIP-1α may provide an additional mechanism to protect cells against the R5-tropic virus during early HIV infection *in vivo*. The consequences of IL-6 and IL-10 increase upon treatment with EboGPΔM-V3 VLPs (Fig. 3B and C) still need more investigation. However, a previous report indicated that simultaneous neutralization of both endogenous IL-6 and IL-10 led to increased inhibition of Ig secretion by B cells triggered by CD40 Ag and AgR (53). Additionally, it has been reported that IL-6 has a Th2-like promoting capacity and can promote the production of Th2-type cytokines (54). Therefore, further investigation is required to elucidate whether there is any functional association between stronger EboGPΔM-V3-induced Th2 cytokine production and more efficient anti-HIV humoral responses. Additionally, more studies are required to investigate whether the presence of EboGPΔM-V3 could enhance broad anti-HIV CTL responses, which is critical to contribute to the reduction in viral loads and the elimination of productively infected cells.

Accumulating evidence has revealed that neutralizing antibodies not only play an essential role in preventing HIV infections but may also help the cellular arm of the immune response prevent or delay the progression to AIDS. During natural infection, neutralizing antibody levels are generally low and isolate specific, although a large amount of antibodies is produced. Although many strategies are being pursued, it is still challenging to elicit antibodies with the desired level of broadly neutralizing ability (55–57) as a result of viral evolution and the immunodominance of Env variable elements or nonneutralizing epitopes. It is well known that many neutralizing epitopes are conformation dependent. Meanwhile, some reports also indicate that inducing neutralizing antibodies can be achieved by the synthesizing peptides representing HIV V3 regions (58). Therefore, the V3 polypeptide (134 aa) in EboGPΔM-V3 could also induce neutralizing antibodies against HIV infection. We will conduct investigations on whether the expression of EboGPΔM-V3 in vaccine preparations such as rVSVΔG/Env/EboGPΔM/V3 (Fig. 6) could induce neutralizing antibodies by itself and enhance levels of neutralizing antibodies against HIV Env. We hope that this vaccination strategy has the potential to aid the design of immunogens to effectively elicit broadly neutralizing antibodies by inserting peptides covering the neutralizing epitopes of HIV Env into EboGPΔM. In such a way, the neutralizing epitopes of Env will be effectively exposed to the host immune system, including DCs and MDMs, and induce high levels of neutralizing antibodies.

A replicating rVSV vector system has emerged as a vital vaccine technology against complex multimeric viral envelope glycoproteins. Accumulating studies have shown that VSVΔG chimeras are a unique and effective technology for immunizing against viral glycoproteins and merit careful investigation and development as an Env spike delivery platform (59–62). Moreover, the Vero cell line is an FDA-approved cell line to produce viral vaccine preparations for clinical use (63). Interestingly, our study revealed that EboGPΔM-V3 fusion protein-incorporating VLPs and rVSVΔGs had efficient cell entry abilities in Vero cells (Fig. 2C and 6B), indicating the feasibility of using Vero cells with this vaccine technology for vaccine production. This study also clearly showed that rVSVΔG-Henv/EboGPΔM-V3 immunization induced significantly higher immune responses than immunization with rVSV-Henv/VSVG (Fig. 6C).

Taken together, the results of our study demonstrate the feasibility of developing a novel EboGP mucin-like domain replacement system as a DC-targeting vaccine technology, and this technology will provide an opportunity to present large heterologous polypeptides derived from different high-risk pathogens, including HIV-1, and elicit efficient specific immune responses. Notably, the rVSV-EboGP vaccine has been proven to be safe and to effectively confer protection against EBOV in human clinical trials (14, 64). Additionally, EboGPΔM-based DC-targeting vaccine technology can be easily adapted into the rVSV vaccine system; therefore, it is feasible to rapidly develop a new and efficient vaccine against specific viral pathogens, including HIV-1.

## MATERIALS AND METHODS

The HIV-1 RT/IN/Env tri-defective proviral plasmid containing a *Gaussia* gene (ΔRI/ΔE/GLuc), the HIV Env glycoprotein-expressing plasmids pLET-Lai (X4 tropic) and pLET-JRFL (M tropic), the codon-optimized Zaire Ebola virus glycoprotein (EboGP)-expressing plasmid (pCAGGS-EboGP), and the mucin-like domain (MLD) deletion-containing EboGP plasmid (pCAGGS-EboGPΔM) were described previously (16). To construct pCAGGS-EboGPΔM-V3 and pCAGGS-EboGPΔM-V3-V5, the PCR-amplified cDNAs encoding HIV Env glycoprotein from amino acid (aa) 233 to 367 (134 aa) or from aa 233 to 473 (241 aa) were inserted into pCAGGS-EboGPΔM at the MLD region (Fig. 1A and B). To construct rVSV coexpressing HIV full-length Env glycoprotein and EboGPΔM-V3 fusion protein(s), we first inserted the cDNA encoding a full-length Env glycoprotein into a VSV-G deletion-containing rVSV vector. Consequently, the cDNA encoding EboGPΔM fusion protein or VSV-G protein was placed at the 3′ end of the HIV Env gene, and the constructed vectors were named rVSVΔG-Henv/EboGPΔM-V3 and rVSVΔG-Henv/VSVG (Fig. 6A).

### Cells, antibodies, and chemicals.

Human embryonic kidney 293T cell, Vero E6 cells, C8166 T cells, and THP-1 cells were cultured in Dulbecco’s modified Eagle medium (DMEM) or RPMI 1640 medium supplemented with 10% fetal bovine serum (FBS). To obtain human MDMs and MDDCs, the CD14^+^ monocytes were isolated from human peripheral blood mononuclear cells (PBMCs) derived from healthy donors by using an Easy Sep II human CD14 positive selection kit (Stemcell Technologies) and were treated with macrophage colony-stimulating factor (M-CSF) or granulocyte-macrophage colony-stimulating factor (GM-CSF)–IL-4 (R&D Systems) for 7 days. The HIV-1 gp120 monoclonal antibody (ID6), HIV-1 gp120 recombinant proteins (IIIB, AE.A244D11, and C.1086D7), peptide gp120 Z3 405–423, peptide gp120 CDC42 419–433, and HIV-1 NA consensus V3 cyclic peptide were obtained from the NIH AIDS Research and Reference Reagent Program. The anti-HIV p24 antibody was previously described (65). Monoclonal antibody (MAb) 42/3.7 against Ebola virus GP was kindly provided by Ayato Takada, Hokkaido University, Japan (66).

### Production and characterization of HIV VLPs and rVSVs.

To produce VLPs containing EboGP-based chimeric fusion protein, 293T cells were cotransfected with package plasmid Δ8.2 and HIV Env (T/M tropic)-, EboGPΔM-, EboGPΔM-V3-, or EboGPΔM-V3-V5-expressing plasmids. For checking the virus entry ability, the HIV ΔRI/ΔE/GLuc^+^ vector was also included in transfection (35). At 48 h of posttransfection, supernatants from transfected cells were clarified by centrifugation at 3,000 rpm for 15 min. VLPs were pelleted by ultracentrifugation and resuspended in endotoxin-free PBS (EMD Millipore Corp.). The HIVGagp24 levels in VLP stocks were quantified by ELISA and kept at −80°C for both *in vitro* infection and *in vivo* immunization experiments.

To examine the levels of incorporation of EboGPΔM and HIV Env V3 or V3-V5 protein in the virus, the purified VLPs were lysed and analyzed by SDS-PAGE and WB with mouse anti-ZGP (42/3.7), anti-HIV gp120 (ID6), anti-HIV gp120 V3 (5F7), or anti-HIV p24 antibodies. To test the virus entry ability of different VLP stocks, equal amounts of EboGP, EboGPΔM, EboGPΔM-V3, EboGPΔM-V3-V5, or HIV Env-GLuc^+^ VLPs (as adjusted by HIV Gagp24) were used to infect CD4^+^ C8166 T cells, TZM-b1 cells, Vero E6 cells, THP-1 cells, MDMs, and MDDCs. At different points postinfection, supernatants were collected and analyzed by GLuc activity or p24 ELISA.

Two rVSV vectors, including rVSV coexpressing HIV envelope glycoprotein and EboGPΔM-V3 (rVSVΔG/HIVEnv/EboGPΔM-V3), were constructed by inserting HIV Env cDNA at the MluI and SphI sites and EboGPΔM-V3 cDNA (or VSV-G cDNA) into the XhoI and NheI sites in the rVSVΔG vector (Fig. 6A). Then, these rVSV vectors were rescued in Vero E6 cells by using a reverse genetics technique (47), and the rescued replicating rVSV viruses were further amplified and concentrated in Vero E6 cells. Finally, they were titrated in Vero E6 cells and used in mouse immunization experiments.

### Analysis of cytokine gene expression *in vitro* by quantitative real-time PCR.

Human MDMs were treated with HIV Env or EboGPΔM-V3 VLPs, and the cells were collected at 4 and 24 h after treatment. Meanwhile, LPS treatment was included as a control. The total cellular mRNA was extracted using a High Pure RNA isolation kit (Roche Life Science) and reverse transcribed into cDNA with Moloney murine leukemia virus (M-MLV) reverse transcriptase (Promega). The synthesized cDNA was subjected to qPCR using LightCycler 480 SYBR green I master mix (Roche Life Science) and cytokine gene-specific primers. IL-2 primers were ATGCCCAAGAAGGCCACAGA (forward) and 3′-GCTGTCTCATCAGCATATTCACAC (reverse). IL-6 primers were 5′-AGCCAGAGCTGTGCAGATGA (forward) and 3′-GCAGGCTGGCATTTGTGGTT (reverse). IL-10 primers were 5′-AAGGCGCATGTGAACTCCCT (forward) and 3′-CCACGGCCTTGCTCTTGTTTT (reverse). TNF-α primers were 5′-AGGCGGTGCTTGTTCCTCA (forward) and 3′-GGCTACAGGCTTGTCACTCG (reverse). MIP-1α primers were 5′-CATGGCTCTCTGCAACCAGTTCT (forward) and 3′-GCCGGCTTCGCTTGGTTAGG (reverse). IFN-γ primers were 5′-CCAGAGCATCCAAAAGAGTGTGG (forward) and 3′-TGGCGACAGTTCAGCCATCA (reverse). The program was 95°C for 10 min (1 cycle); 95°C for 10 s, 55°C or 60°C for 20 s, and 72°C for 30 s (40 cycles); and 72°C for 2 min (1 cycle). The fold change in mRNA levels in treated cells was calculated compared to mock-treated controls after normalizing to the GAPDH gene.

### GLuc assay and NF-κB activity luciferase reporter assay.

The *Gaussia* luciferase (GLuc) assay was done as follows. At various time points after infection, supernatants from the cell cultures were collected. A 50-μl portion of GAR-1 reagent (Targeting Systems) was added to 10 μl of sample, mixed well, and then measured in the luminometer (Promega, USA) (35). For the NF-κB activity luciferase reporter assay, the VSV-G-pseudotyped lentiviral particles (Cignal Lentivector) expressing a reporter firefly luciferase gene under the control of a minimal cytomegalovirus (CMV) promoter and tandem repeats of the NF-κB transcriptional response element (TRE) (catalog no. 336851; Qiagen, Hilden, Germany) were used to transduce THP1 cells, a monocyte-like cell line (67). Following transduction, the THP1 cells were cultured under puromycin selection to generate a homogenous population of the THP1–NF-κB sensor cell line. Then, the THP1–NF-κB sensor cells were treated with EboGPwt, EboGPΔM-V3, or HIV Env VLPs or TNF-α up to 18 h, lysed, and subjected to a luciferase assay to monitor NF-κB signaling activity upon various VLP treatments (35).

### Mouse immunization experiment.

Female BALB/c mice, aged 4 to 6 weeks, were obtained from the Central Animal Care Facility of the University of Manitoba (with the animal study protocol approval no. 16-012/1/2 [AC11159] by the University of Manitoba Bannatyne Campus Animal Care Committee). The mice (four mice per group) were injected subcutaneously with 100 ng (HIV p24) of various VLPs in 100 μl endotoxin-free PBS on day 0 and given boosters on days 21 and 49 or only on day 29. Mice were sacrificed at day 56 or 42. Blood samples were obtained at different time points (Fig. 4 and 5B). For rVSV immunization, mice were immunized with 1 × 10^6^ TCID_50_ of rVSV once and sacrificed on day 35. Blood samples were obtained on days 7, 27, and 35. All blood samples collected were allowed to clot for 30 min to 1 h at room temperature and were centrifuged at 1,500 × *g* for 10 min at 4°C. The resulting sera were stored at −20°C until further analysis. Vaginal wash samples were collected by washing with 100 μl of sterile PBS, delivered, and recovered five consecutive times on the sacrifice day.

### Anti-HIV antibody measurements by ELISA.

To measure the HIV Env- and Gag-specific antibodies in sera, ELISA plates (Nunc MaxiSorp, Thermo Scientific) were coated with 100 μl of HIV-1 gp120 recombinant protein (IIIB, AE.A244D11, or C.1086 D7gp120) in 1 μg/ml or HIV-1 IIIB p24 recombinant proteins (0.5 μg/ml) as described previously (16). After the incubation of diluted mouse serum (1:100 to 1:500) samples in coated ELISA plates, peroxidase-conjugated goat anti-mouse immunoglobulin G (IgG) (GE Healthcare) or IgA (Invitrogen) was used as the secondary antibody. To test whether the antibodies from immunized mice can recognize the specific regions of HIV-1 gp120, the ELISA was also processed by coating the HIV-1 consensus B V3 peptide (CKSIHIGPGRAFYTTGC) or the peptides of HIV Env from aa 405 to 423 and aa 419 to 433, as described above.

To detect HIV-specific gp120 or p24 IgG or IgA in vaginal wash fluids, horseradish peroxidase (HRP)-conjugated anti-mouse IgG or IgA (Invitrogen) was used after the incubation of diluted mouse vaginal wash samples (1:50 dilution) in HIV-1 gp120 or p24 recombinant protein-coated 96-well plates.

### Cytokine detection.

Splenocytes from immunized mice were placed in a cell strainer, and the plunger end of the syringe was used to mash the spleen through the cell strainer into the petri dish to make single-cell suspensions. Suspensions were cultured in 48-well plates at a density of 2 × 10^6^/125 μl with DMEM containing an HIV-1 PTE (potential T cell epitope) Env peptide pool (3 μg/peptide/ml). Supernatants were harvested following 3 days of culture and stored at −70°C until the assay. Cytokine levels (IFN-γ, IL-2, IL-4, IL-5, and MIP-1a) were quantified using the mouse MSD V-plex kit (Mesoscale Discovery; USA). Mean lower limits of detection were as follows: IFN-γ, 0.186 pg/ml; IL-2, 0.617 pg/ml; IL-4, 0.437 pg/ml; IL-5, 0.242 pg/ml; and MIP-1α, 0.18 pg/ml.

### Statistical analysis.

Statistical analysis of levels of gp120, p24 in the ELISA, and expression of various cytokine genes was performed using the unpaired *t* test (considered significant at a *P* value of ≤0.05) by GraphPad Prism 6.01 software.

### Data availability.

The data sets generated and/or analyzed during the current study are available from the corresponding author.
